# Chemical communication and host search in *Galerucella* leaf beetles

**DOI:** 10.1007/s00049-014-0174-1

**Published:** 2014-10-18

**Authors:** Lisa Fors, Ilme Liblikas, Petter Andersson, Anna-Karin Borg-Karlson, Nancy Cabezas, Raimondas Mozuraitis, Peter A. Hambäck

**Affiliations:** 1 Department of Ecology, Environment and Plant Sciences, Stockholm University, 106 91 Stockholm, Sweden; 2 Institute of Technology, University of Tartu, Tartu, 50411 Estonia; 3 Department of Chemistry, KTH Royal Institute of Technology, 100 44 Stockholm, Sweden; 4 Laboratory of Chemical and Behavioural Ecology, Institute of Ecology, Nature Research Centre, Akademijos 2, 08412 Vilnius, Lithuania

**Keywords:** Pheromone, Volatiles, Plant–herbivore interactions, Olfactometer

## Abstract

Herbivore insects use a variety of search cues during host finding and mate recognition, including visual, gustatory, and olfactory stimuli, leaving multiple traits for evolution to act upon. However, information about differences or similarities in search pattern amongst closely related insect herbivore species is still scarce. Here, we study the production of and the response to pheromone in *Galerucella* (Coleoptera: Chrysomelidae) to investigate the beetles’ search behaviour. Males of *G*. *pusilla* and *G. calmariensis*, two closely related species, are known to produce the aggregation pheromone dimethylfuran-lactone when feeding on their host plant, whereas no pheromones have been identified in other *Galerucella* species. We show that dimethylfuran-lactone is produced also by males of *G*. *tenella*, a species phylogenetically close to *G*. *pusilla* and *G*. *calmariensis*, whereas the more distantly related species *G. lineola* and *G. sagittariae* were not found to produce the same compound. To investigate the beetles’ behavioural response to dimethylfuran-lactone, the pheromone was synthesized using a partly novel method and tested in olfactometers, showing that *G*. *pusilla*, *G*. *calmariensis*, and *G*. *tenella* were all attracted to the pheromone, whereas *G*. *lineola* and *G*. *sagittariae* did not respond. This suggests that the production of and the response to pheromone could be linked to the phylogenetic relatedness between the species.

## Introduction

Closely related species of insect herbivores must evolve means both of mate recognition and of separating current and ancestral host plant species. In some cases, mating occurs on the plant and the two processes interact, as in many chrysomelid species. Host search and mate finding involve visual, gustatory, and olfactory cues (Schoonhoven et al. 2005), leaving multiple traits for evolution to act upon. Nevertheless, there is limited information on the similarities or differences amongst closely related herbivore species in search cues used during the host-finding process. Host shifts in insect herbivores typically occur onto closely related plant species, which often produce similar volatile blends as the original host (Bengtsson et al. 2006). On the other hand, more generalist herbivore species may use host plants of quite distant relationships, which further complicates the inclusion of additional host plant species in the diet repertoire.

There may however be feedback mechanisms that more efficiently connect host and mate finding, when either males or females produce pheromones that attract conspecifics. Whilst individuals may not necessarily reside on the host plant when emitting pheromone, there are several cases when prior feeding on the host plant is a prerequisite for pheromone production (Reddy and Guerrero 2004; Saïd et al. 2011). In chrysomelid and curculionid beetles, male-produced pheromones seem common (Dickens et al. 2002; Cossé et al. 2005; Zilkowski et al. 2006; Ambrogi et al. 2009), and these pheromones may attract both males and females and in some cases also closely related species. For instance, males of both *Galerucella calmariensis* and *G. pusilla* produce the same pheromone and there is also interspecific attraction to beetle-damaged plants (Bartelt et al. 2008; Hambäck 2010). In addition to mate attraction, there are several benefits associated with pheromone emission. In some species, larval growth or survival is enhanced when individuals are aggregated (Hunter 2000; Allen 2010). If herbivorous insects occur at a high density they are more prone to break the defence of the host, a process that is particularly well known for many forest pests, where large aggregations may cause massive plant death (Berryman et al. 1989; Hunter 2000). Aggregations can also be a way to avoid or minimize potential threats. When aggregated, the risk of being attacked by natural enemies, such as predators, parasites or parasitoids, is lower for each individual (Codella and Raffa 1995; Morris et al. 1996). On the other hand, natural enemies can take advantage of the aggregating behaviour and follow the same odour cues as the herbivores to find suitable hosts (Dicke and van Loon 2000; Raffa et al. 2007; Girling et al. 2011).

**Fig. 1:**
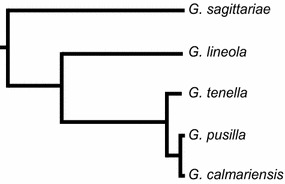
Phylogeny of *Galerucella* spp. included in this study. Branch lengths are correlated to evolutionary distance between the species

In this study five species of *Galerucella* (Coleoptera: Chrysomelidae) leaf beetles were used to investigate the production of and the response to pheromone. Males of *G*. *pusilla* and *G*. *calmariensis*, two closely related species, have been shown to produce the aggregation pheromone dimethylfuran-lactone when feeding on their host plant *Lythrum salicaria* (Bartelt et al. 2006), whereas no pheromones have been identified in other *Galerucella* species. This study quantified pheromone production in three species at different phylogenetic distances from *G. pusilla* and *G. calmariensis* (Fig. 1). In addition, we compared behavioural responses amongst the five species to the pheromone alone and in combinations with host plant odours. As *G*. *calmariensis* and *G*. *pusilla* only produce the pheromone when feeding on the host plant, we hypothesized that attraction was either restricted to or stronger in the presence of host plant odours.

## Materials and methods

### Study species

Five species of *Galerucella* leaf beetles (Coleoptera: Chrysomelidae): *Galerucella calmariensis* (L.), *G. pusilla* (Duftschmid), *G. tenella* (L.), *G. sagittariae* (Gyllenhaal) and *G. lineola* (Fabricius) are included in this study (Fig. 1). The beetles over-winter as adults and emerge during spring, when mating takes place on the host plants. The eggs are deposited directly on the leaves or stem in early summer and hatch after a few weeks. Both larvae and adults feed on the plant, which can sometimes lead to quite severe damage. After 2–4 weeks of larval feeding the larvae pupate and the new adults emerge from the pupae 2–3 weeks later (Hambäck 2004). Both *G*. *pusilla* and *G*. *calmariensis* use *L*. *salicaria* (Lythraceae) exclusively as host plant, for feeding and oviposition. *G. lineola* uses species of Salicaceae and *Alnus* (Betulaceae) as hosts, whereas *G. tenella* primarily uses *Filipendula* *ulmaria* (Rosaceae) and other Rosaceae species, including *Fragaria* sp. *G. sagittariae* also uses *Fragaria* sp. as host plants, as well as other Rosaceae and Primulaceae species (Hambäck et al. 2013). For the pheromone collection experiments, adults of *G*. *calmariensis*, *G. pusilla*, *G. tenella*, *G. sagittariae*, and *G. lineola* were collected in May and June 2012 from various localities in the counties of Västerbotten, Västernorrland, Gävleborg, and Uppsala (Sweden). For the behavioural studies, adults of all beetle species were collected during May and June 2013 from various localities in the counties of Uppsala and Gävleborg (Sweden). Before trials, the beetles were stored in ventilated plastic boxes in the laboratory and fed with fresh leaves from their respective host plant when needed. All plants used were potted and kept in the common garden or green house prior to the tests.

### Collection of pheromone

Bartelt et al. (2006, 2008) have reported that male beetles of both *G*. *pusilla* and *G*. *calmariensis* produce dimethylfuran-lactone only when feeding on their host plants. Hence, all five species of *Galerucella* included in this study were used with one of their respective host plants: *G. calmariensis* and *G. pusilla* with *L. salicaria*, *G. tenella* and *G. sagittariae* with *Fragaria x ananassa*, and *G. lineola* with *Salix viminalis*. The pheromone collection was performed from late May to early July 2012. A few days before the tests, all plants were checked thoroughly for any herbivorous insects and kept in the green house until the time for the experiments. Plants in bloom or plants afflicted by herbivores or showing fresh cuts or injuries were not used in the experiments.

To distinguish volatiles emitted by the beetles from those released by the plants, three experimental setups were used for each beetle–host plant pair: (1) 10 male beetles per plant [*G*. *calmariensis* (*n* = 3 × 10), *G. pusilla* (*n* = 3 × 10), *G. tenella* (*n* = 4 × 10), *G. sagittariae* (*n* = 3 × 10) and *G. lineola* (*n* = 2 × 10)]; (2) 10 larvae (third instar) per plant (*n* = 6 × 10 for all species) and (3) mechanically damaged plants without beetles or larvae [*n* equals the number of plants used in (1) for each species]. The leaves of the mechanically damaged plants were perforated using a preparation needle (1.5 mm diameter) prior to volatile collection. As far as possible, the same amount of damage was inflicted to each plant, in total 100 perforation holes per plant. This roughly mimicked the amount of damage inflicted by 10 feeding beetles during the time for volatile collection (24 h). Approximately the same leaf biomass was used for each setup and a new plant was prepared for each experiment.

The plant part used (either alone or bearing beetles, larvae or 1 cm^2^ filter paper with 1 μg pentadecane as internal standard) was enclosed in a polyester cooking bag (Toppits, 25 × 40 cm) and volatiles were collected during 24 h using a solid phase micro-extraction (SPME) technique (Pawliszyn 1997). Important considerations have to be taken into account regarding the use of SPME technique for collection of constantly produced volatiles and interpretation of data. For this purpose, volatiles were collected and analysed in exactly the same way, using the same fibre type, the same headspace volume, controlled, stable temperatures during sampling and addition of internal standard to monitor unsaturation of fibres. Under these circumstances, SPME is an extremely sensitive, user-friendly, and excellent technique. However, comparison of quantities of different compounds in the same sample without the use of labelled standards is not possible because of different affinities of compounds on the fibre and because of different vapour pressures of the target compounds (based on SPME guidelines, J Chem Ecol). SPME fibres (Supelco, Sigma-Aldrich group, PA, USA) coated with 65 µm polydimethylsiloxane-divinylbenzene were selected based on the literature data (Vas and Vekey 2004) and personal experience. The routine purification of the SPME fibre was done at 225 °C for about 2 min in a gas chromatograph (GC) injector before each odour sampling. After purification of the fibre, the wall of the polyester bag was pierced by the needle of the syringe, and the purified fibre was exposed to a headspace. The male beetles or larvae were put on the plant just before enclosing it in the polyester bag and allowed to feed during the time for volatile collection (24 h). After collection, the fibre was retracted into the needle, removed from the bag and placed into a GC injector for desorption of volatiles.

### Chemical analyses

The samples were analysed using a Varian 3400 GC (Varian, Palo Alto, CA, USA) coupled with a Finnigan SSQ 7000 mass spectrometer (MS) (Termo-Finnigan, San Jose, CA, USA). A DB-wax silica capillary column (J&W Scientific, Folsom, CA, USA, 30 m, internal diameter 0.25 mm, film thickness 0.25 µm) was used with a temperature program of 40 °C (1 min), increasing 5 °C/min to 200 °C, and subsequently by 10 °C/min up to 230 °C, and thereafter held isothermally at 230 °C for 15 min. The split/splitless injector temperature was 225 °C and the splitless period was 30 s. Helium was used as the carrier gas with an inlet pressure of 10 psi. Electron ionization mass spectra were determined at 70 eV with the ion source at 150 °C in the range 30–400 Daltons. TIC (Total Ion Chromatograms) obtained from the plants with feeding *Galerucella* beetles were screened for compounds structurally related to the known aggregation pheromone, dimethylfuran-lactone, using diagnostic ions *m/z* 135, *m/z* 149, *m/z* 193, *m/z* 208, and *m/z* 236. Identity of dimethylfuran-lactone in the samples was confirmed by comparison of mass spectral data and GC-retention times of natural product with the corresponding data of synthetic standard and those published by Bartelt et al. (2006). Relative quantities of methylfuran-lactone were determined by combining indicated diagnostic ions at the range ±0.5 Dalton into a single chromatogram to decrease noise level. Relative amount of the target compound was expressed as an area under a peak using arbitrary units.

### Pheromone synthesis

**Fig. 2:**
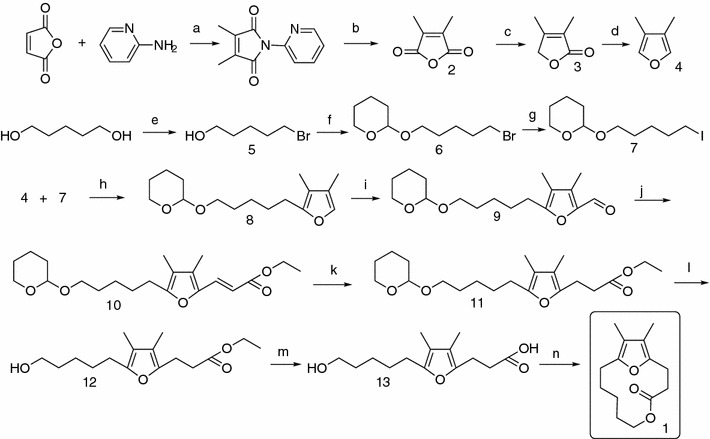
Synthetic scheme of *Galerucella* pheromone dimethylfuran-lactone: 12,13-dimethyl-5,14-dioxabicyclo[9.2.1]tetradeca-1(13),11-dien-4-one (**1**). Reagens and conditions used in the various steps *a* AcOH, 120 °C; *b* H_2_SO_4_, 100 °C; *c* NaBH_4_, THF, 0 °C; *d* DIBAL, Et_2_O, −40 °C; *e* 48 % HBr, toluene, reflux; *f* DHP, DCM, Amberlyst 15; *g* NaJ, Acetone; *h* *n*-BuLi, THF, −20 °C, *i* *n*-BuLi, THF, DMF, −20 °C, *j* TEPA, LiO*t*Bu, hexane, 25 °C; *k* 10 % Pd/C, H_2_, hexane, rt; *l* Amberlyst^15^, MeOH, 50 °C; *m* KOH, MeOH/H_2_O (1:1), 45 °C; *n* DEAD, Ph_3_P, toluene, rt

The synthetic scheme of the *Galerucella* spp. pheromone 12,13-dimethyl-5,14-dioxabicyclo[9.2.1]tetradeca-1(13),11-dien-4-one (1) is presented in Fig. 2. The synthetic pathway to the dimethylfuran-containing macrolide pheromone **1** consists of preparing 3,4-dimethylfuran **4**, attaching suitable hydroxyl and acyl side chains at position 2 and 5, and finally closing the lactone cycle. This synthesis follows the main part the paths described by Bartelt et al. (2006) and Petroski et al. (2009), but differs in how to make 3,4-dimethylfuran, for which better, less sluggish reactions were developed. Detailed descriptions of the synthetic pathways and structure elucidations are found in “Appendix”.

### Behavioural study

Behavioural responses of *Galerucella* spp. to plant odours and pheromone were studied in two-armed olfactometers from late May to early July 2013. The olfactometers comprised three layers of acrylic glass with an arena cut out in the middle layer, consisting of a central zone with two tapered arm zones (Hambäck et al. 2003). A vacuum pump was connected via Teflon tubes at the top of the olfactometer and the airflow was adjusted to draw room-tempered, non-humidified air through the arena at a constant rate of approximately 3 ml/s. This setup was arranged so that four olfactometer trials could be run simultaneously. Before trials, one beetle was allowed to acclimatize inside the olfactometer for 5 min. Subsequently, notes were taken on the beetle’s position in the arena at 1-min intervals for 30 min, giving a total of 30 recordings in the arena for each beetle individual. After the trials, the sex of each beetle was determined through dissection of the genital parts. Beetles that were inactive for more than 5 min during the trials were excluded from the study. Between the trials, the olfactometers were washed with water and a mild detergent and thereafter rinsed in 70 % ethanol, and the positions of the syringes connected to the olfactometer arms were switched.

The olfactometer studies were started by investigating the behavioural responses of *G*. *calmariensis*, *G. pusilla*, *G. tenella*, *G. sagittariae*, and *G. lineola* to the synthetic pheromone (dimethylfuran-lactone). Each arm zone of the olfactometer was connected to a 100-ml plastic syringe, with the plunger withdrawn to allow air to be drawn through the arena. In each syringe barrel a small rubber dispenser was placed (washed in methylene-chloride prior to the experiment); one was loaded with 100 μg dimethylfuran-lactone diluted in 50 μl hexane and the other was loaded with 50 μl hexane, serving as a control.

Next, the behavioural responses of the beetles to a blend of the pheromone and odours from their respective host plants were investigated. These studies were performed on *G. pusilla* with *L*. *salicaria* as host plant and *G. tenella* with *F*. *ulmaria* as host plant, as these species (and *G. calmariensis*) responded to the pheromone in the first experiment (see results). *G. calmariensis* was not included in this part of the test as the number of individuals was too low for meaningful analyses. The experimental procedure followed the protocol described above, but with different odour treatments; (1) pheromone vs. a blend of pheromone/host plant odour and (2) host plant odour vs. a blend of pheromone/host plant odour. The amount of dimethylfuran-lactone was the same in all three pheromone treatments (100 μg dimethylfuran-lactone diluted in 50 μl hexane). For the host plant treatments 10- to 15-cm branches were cut from potted plants of *L*. *salicaria* and *F*. *ulmaria* and inserted into the syringes. Plants in bloom or plants afflicted by herbivores were not used in the tests. The branches were cut so that each branch had on average 5–7 leaves to keep the leaf biomass similar amongst the trials. As *Galerucella* beetles usually respond stronger to damaged plants compared to non-damaged plants, five leaves on each branch were mechanically damaged (one hole per leaf) using a preparation needle (1.5 mm diameter).

### Statistical analyses

Relative amounts of dimethylfuran-lactone trapped from male beetles of the five *Galerucella* species, as well as those of the internal standard, were log transformed and analysed for variance using ANOVA. Zero values were not included in the analyses. ANOVA revealed differences in relative amounts of dimethylfuran-lactone; hence, Tukey HSD test for unequal N was used to determine the significance of the differences using an experiment-wise error rate set at *α* = 0.05. The analyses were carried out using the computer program package Statistica, version 12. In the statistical analyses of the behavioural data, the replicate was the individual beetle and the analyses were performed on the number of recordings each beetle individual spent in each arm zone during the 30-min session. In the first part of the behavioural test (responses to the synthetic pheromone) all five *Galerucella* species were included and in the second part (involving host plant odours) *G. pusilla* and *G. tenella* were included. Wilcoxon signed rank tests were used to analyse whether the beetles visited one arm zone significantly more than the other. Recordings made when the beetles were located in the central zone of the arena were excluded, as these recordings provided less information about attraction towards any stimulus. Analyses of behavioural data were performed using R 2.15.2 (R Development Core Team 2012).

## Results

### Collection of pheromone

**Fig. 3:**
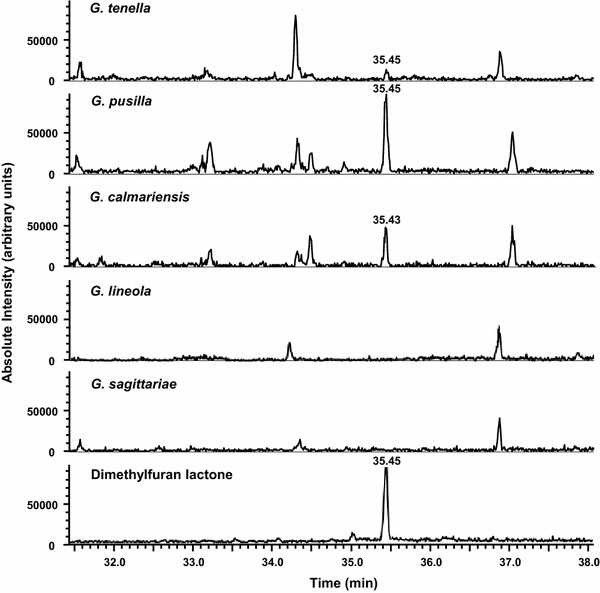
Selected ion chromatogram of records of volatiles collected from plants with feeding *Galerucella* beetles as well as from synthetic dimethylfuran-lactone. The chromatogram was displayed using the fragments *m/z* 135, *m/z* 149, *m/z* 193, *m/z* 208, and *m/z* 236 at the range ±0.5 Dalton to decrease noise level; DB-Wax fused silica capillary column (30 m length, 0.25-mm ID, 0.25-µm film thickness)

**Fig. 4:**
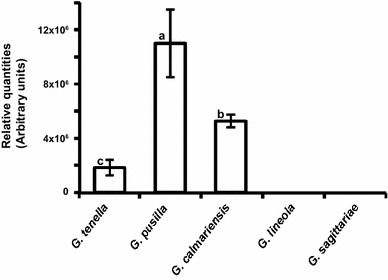
Relative quantities (±SE) of dimethylfuran-lactone obtained from feeding *Galerucella* beetles. The values on the *Y* axis are quantities (*peak areas*) corresponding to the amount of compound analysed, related to the abundance of ions formed. Values indicated by different *letters* are significantly different (Tukey HSD test for unequal *N*, *p* < 0.05)

The relative amounts of the internal standard, pentadecane, trapped on the fibres and injected on the column did not differ significantly for any of the samples (ANOVA, *F* = 1.73, *p* = 0.23), indicating that saturation of the fibres was not reached. The aggregation pheromone (dimethylfuran-lactone) of *G. calmariensis* and *G. pusilla*, known from earlier studies (Bartelt et al. 2006), was found also in our experiments, emitted from feeding males of these two species (Fig. 3). The same pheromone was produced in significantly lower amounts also by males of *G. tenella* (ANOVA *F* = 13, *p* = 0.004) (Figs. 3, 4), whereas there was no trace of this pheromone in *G. lineola* and *G. sagittariae* (Fig. 3). In the experiment using larvae instead of adult beetles, no compounds were found that could be distinguished from the volatiles emitted from the plants.

### Behavioural study

**Fig. 5:**
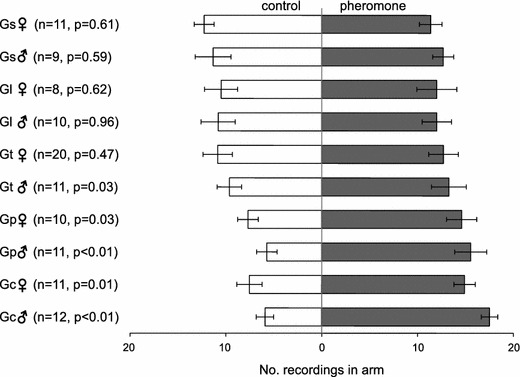
Behavioural responses in *Galerucella* spp. to synthetic pheromone, dimethylfuran-lactone (Gs = *G. sagittariae*, Gl = *G*. *lineola*, Gt = *G. tenella*, Gp = *G. pusilla*, Gc = *G*. *calmariensis*). Data were analysed using Wilcoxon signed rank tests. *Error bars* indicate standard error of the mean

Differences were found amongst the five *Galerucella* species in their behavioural responses to the synthetic pheromone (dimethylfuran-lactone). Similar to previous studies, both males and females of *G. calmariensis* and *G. pusilla* were recorded significantly more often in the arm zone providing the pheromone compared to the control arm zone providing hexane [*G. calmariensis* ♂: (*n* = 12, *p* < 0.01) and ♀: (*n* = 11, *p* = 0.01), *G. pusilla* ♂: (*n* = 11, *p* < 0.01) and ♀: (*n* = 10, *p* = 0.03)] (Fig. 5). Males of *G. tenella* were recorded significantly more often in the arm providing the pheromone (*n* = 11, *p* = 0.03), suggesting an attraction to the pheromone, whilst the females did not show any attraction (*n* = 20, *p* = 0.47) (Fig. 5). There was no evidence for *G. sagittariae* or *G. lineola* to be attracted to the pheromone [*G. sagittariae* ♂: (*n* = 9, *p* = 0.59) and ♀: (*n* = 11, *p* = 0.61), *G. lineola* ♂: (*n* = 10, *p* = 0.96) and ♀: (*n* = 8, *p* = 0.62)] (Fig. 5).

**Fig. 6:**
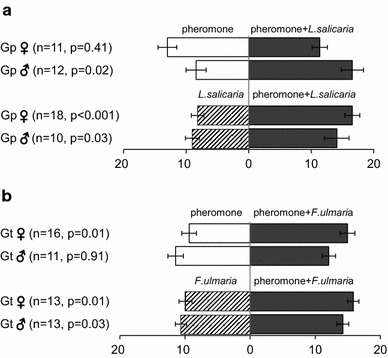
Behavioural responses of **a** *G. pusilla* (Gp) and **b** *G. tenella* (Gt) to blends of synthetic pheromone (dimethylfuran-lactone) plus host plant odours vs. either pheromone alone or host plant odour alone. Data were analysed using Wilcoxon signed rank tests. *Error bars* indicate standard error of the mean

In the behavioural studies involving host plant odours, males of *G. pusilla* visited the arm zone providing a blend of the pheromone and *L. salicaria* odour significantly more often than the arm zone with only the pheromone (*n* = 12, *p* = 0.02), suggesting that the males were attracted to the blend, whilst the females did not discriminate between the treatments (*n* = 11, *p* = 0.41) (Fig. 6). When given a choice between the blend and *L. salicaria* odour only, both sexes showed a significant attraction to the blend [♂: (*n* = 10, *p* = 0.03) and ♀: (*n* = 18, *p* < 0.001)] (Fig. 6). Somewhat different patterns were observed for *G. tenella*, where the females preferred the blend over the pheromone alone (*n* = 16, *p* = 0.01), whilst the males did not discriminate between the treatments (*n* = 11, *p* = 0.91) (Fig. 6). Moreover, both males and females of *G. tenella* visited the arm zone with the blend significantly more often than the arm zone providing only *F. ulmaria* odour [♂: (*n* = 13, *p* = 0.03) and ♀: (*n* = 13, *p* = 0.01)] (Fig. 6).

## Discussion

Males of the closely related chrysomelid species *G*. *pusilla* and *G*. *calmariensis* are known to produce the pheromone dimethylfuran-lactone that attracts conspecifics (Bartelt et al. 2006, 2008). In the study presented here, the same pheromone is shown to be emitted in lower amounts also by a third species, *G*. *tenella*. When investigating the beetles’ behavioural responses to synthetic pheromone, both sexes of *G*. *pusilla* and *G*. *calmariensis* were attracted to the pheromone. The response for *G*. *tenella* was less distinct, only males were attracted to the compound without host plant odours whilst both sexes were attracted to the pheromone with host plant odours. In *G. lineola* and *G. sagittariae* no production of dimethylfuran-lactone was detected and no attraction to the synthetic pheromone was observed.

In *G*. *pusilla* and *G*. *calmariensis*, dimethylfuran-lactone is only produced when the beetles are feeding on *L*. *salicaria* (Bartelt et al. 2006, 2008), which indicates that a combination of pheromone and green leaf volatiles from the host plant is needed to attract conspecifics. Accordingly, individuals of *G*. *pusilla* and *G*. *tenella* were more attracted to a blend of pheromone and host plant odour compared to host plant odour alone. When comparing the blend to the pheromone alone, females of *G*. *tenella* and males of *G*. *pusilla* were more attracted to the blend. Thus, even though *G*. *tenella* clearly responds to the same pheromone as *G*. *pusilla* and *G*. *calmariensis*, it seems as if both the pheromone and host plant volatiles are of importance at aggregation. One reason for the low pheromone emission found in *G*. *tenella* compared to *G*. *pusilla* and *G*. *calmariensis* could be the choice of host plant for the pheromone collection experiment, where the beetles were feeding on *F*. *x ananassa*. Although *Fragaria* sp. are adequate hosts for *G*. *tenella*, *F*. *ulmaria* is the more common host plant used in the field. There is a possibility that the level of pheromone produced differs when the beetles are feeding on different plant species.

As *G*. *tenella* is the species most closely related to *G*. *pusilla* and *G*. *calmariensis* (Hambäck et al. 2013), it could explain why *G*. *tenella* produces and responds to the same pheromone, even though it uses a different host plant than the other two species. *G*. *lineola*, more distantly related to *G*. *pusilla* and *G*. *calmariensis*, neither produces nor responds to the pheromone. The same is true for *G. sagittariae*, furthest apart in the phylogeny. However, it is possible that males of *G*. *lineola* and *G. sagittariae* produce other pheromones not shown here. Although dimethylfuran-lactone is the only pheromone detected and tested in this study, additional pheromone components may be present. Putative aggregation pheromones in *G. lineola* and *G. sagittariae* are most likely structurally similar to those for the other *Galerucella* beetles. In curculionid beetles, previous studies suggest that the chemical structural relationships of aggregation pheromones are in accordance to the subfamily taxonomy (Ambrogi et al. 2009). However, this is an area that is still poorly investigated. In this study, no compounds have yet been detected that could function as putative pheromone components in *G. lineola* and *G. sagittariae* when investigating the GC–MS data for compounds structurally related to dimethylfuran-lactone. Whether this is due to true absence of volatiles or too low emission of compounds released by the beetles in relation to volatiles released by the plants cannot be assessed in this study.

To conclude, pheromone production and response in *Galerucella* seem to be connected to the phylogenetic relatedness between the beetle species, where the three most closely related species included in this study both produce and respond to the same pheromone (dimethylfuran-lactone). Interestingly, these three species are also attacked by the same parasitoid (*Asecodes parviclava*), whereas the two congeners, *G*. *lineola* and *G. sagittariae*, are attacked by two separate *Asecodes* species. It is still unknown what search cues the parasitoids use to find the right host, but one possibility is that they exploit the beetles’ pheromone as a host cue kairomone. If so, the parasitoid–beetle interactions could clearly affect the beetles’ pheromone composition over time. To better understand the pheromone evolution in *Galerucella*, additional ecological factors apart from reproductive isolation ought to be taken into account. In future studies, evolutionary arms race with natural enemies (such as parasitoids) that could affect changes in the beetles’ pheromone production needs to be considered.

## Figures and Tables

**Fig. 7:**
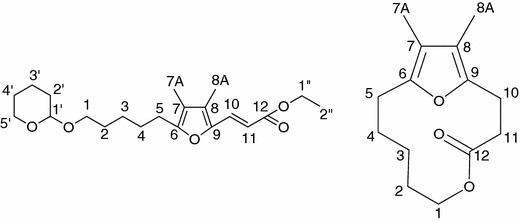
Numbering of the carbons to follow the ^1^H and ^13^C chemical shifts for intermediate compounds and the final product

## Appendix: Synthesis of macrolide pheromone, 12,13-dimethyl-5,14-dioxabicyclo[9.2.1]tetradeca-1(13),11-dien-4-one (1)

The synthetic pathway to dimethylfuran-containing macrolide pheromone **1** consists of preparing 3,4-dimethylfuran **4**, attaching suitable hydroxyl and acyl side chains at position 2 and 5, and finally closing the lactone cycle. Our synthesis follows in many ways the path described by Bartelt et al. (2006) and Petroski et al. (2009), but differs in the making of 3,4-dimethylfuran, for what we found better, less sluggish reactions.

First, we prepared dimethylmaleic anhydride **2** by decarboxylative dimerization of maleic anhydride in the presence of 2-aminophenol. Sodium borohydride reduction of **2** yielded lactone **3**, after which DIBAL reduction gave furan **4**.

THP-protected iodohydrin **7** was prepared from α,ω-pentanediol by monobromination with aq. HBr in toluene, followed by hydroxyl protection with DHP in DCM. Bromine was replaced by iodine with NaI in acetone via a frequently used well-known procedure to yield **7**.

3,4-dimethylfuran **4** was lithiated with *n*-BuLi and alkylated with iodide **7**. The resulting trialkylated furane **8** was lithiated again and thereafter formylated with DMF to yield the aldehyde **9**. The reaction of phosphonate carbanion from triethyl 2-phosphonoacetate, according to Horner–Wadsworth–Emmons procedure, gave the ester **10**. Reduction over Pd-catalyst, deprotection of the alcohol function, and hydrolysis of the ester yielded the furanoic hydroxy acid **13** which was cyclized according to Mitsunobu procedure to form the macrolide **1**.

## Experimental part

The preparative liquid chromatography technique used (MPLC) was the one described by Baeckström et al. (1987). It was performed on silica gel (*Merck* 60, 0.040–0.063 mm) in 15, 35, or 25 mm inner diameter glass columns with gradient elution, using petroleum ether and increasing proportions of ethyl acetate. ^1^H and ^13^C NMR spectra of CDCl_3_ solutions were recorded at 400 and 101 MHz, respectively, using a Bruker AM spectrometer. The tetrahydrofuran (THF) was dried on molecular sieves (4 Å). The other starting materials were purchased from commercial suppliers (Sigma-Aldrich, Fluka) and used without further purification. The reactions involving anhydrous solvents were carried out in an atmosphere of nitrogen.

### 3,4-dimethylfuran-2,5-dione (**2**) (Baumann et al. 1984; Schobert and Barnickel 2009)

The solution of maleic anhydride (58.8 g, 600 mmol) in AcOH (90 ml) was added to a solution of 2-aminopyridine (28.8 g, 300 mmol) in AcOH (60 ml) at 80 °C within 1 h. After refluxing for 3 h, the solvent distilled and aq. 2 M H_2_SO_4_ (180 ml) was added to the residue. The reaction mixture was refluxed for another 2 h. Cooling to rt resulted in precipitation which was filtered and washed with cold H_2_O (3 × 50 ml) to yield **2** (29.4 g, 77.8 %) as off-white crystals after drying.

^1^H NMR (400 MHz, CDCl_3_): *δ* (ppm): 2.05 (s,6H)

^13^C NMR (101 MHz, CDCl_3_): δ (ppm): 166.2, 140.6, 9.2.

### 3,4-dimethylfuran-2(5*H*)-one (**3**) (Abe et al. 1991; Bailey and Johnson 1970; Gogoi and Argade 2006)

To the stirred solution of dimethylmaleic anhydride **2** (19.3 g, 153.2 mmol) in THF (300 ml), NaBH_4_ (10.1 g, 265.8 mmol) was added in portions at 0 °C and stirred on ice bath for 2.5 h. The reaction was quenched carefully (foam!) with H_2_O, acidified with 1 M HCl and extracted with Et_2_O (3 × 150 ml). The combined organic fractions were washed with brine and dried with MgSO_4_. Concentration and purification of the residue using MPLC yielded **3** (15.1 g, 88 %).

^1^H NMR (400 MHz, CDCl_3_): *δ* (ppm): 4.63–4.55 (m, 2H), 1.99 (s, 3H), 1.78 (s, 3H).

^13^C NMR (101 MHz, CDCl_3_): *δ* (ppm): 175.29, 156.23, 123.01, 72.46, 12.12, 8.19.

### 3,4-dimethylfuran (**4**) (Montana et al. 2012; Petroski et al. 2009)

To a solution of **3** (10.5 g, 93.7 mmol) in Et_2_O (250 mL), DIBAL (1.0 M in hexanes, 106 mL) was added at −20 °C over a period of 45 min. Once the addition was complete, the reaction was stirred at −30° to −20 °C for 2 h before it was quenched with H_2_O (5 mL) and 10 % H_2_SO_4_ (60 mL). After warming to an ambient temperature, the aqueous phase was extracted with ether (3 × 40 mL) and the ether extracts were combined with the organic phase, washed with brine (30 mL) and saturated NaHCO_3_ solution (30 mL) and dried over MgSO_4_. Removal of solvent by distillation through a Vigreux column was followed by collection of fraction at 98–103 °C yielded **4** (5.8 g, 64 %).

^1^H NMR (400 MHz, CDCl_3_): δ (ppm): 7.16 (2H), 1.96 (6H).

### 5-Bromo-pentane-1-ol (**5**) (Chong et al. 2000)

To a mixture of 1,5-pentanediol (25.0 g, 240 mmol) and toluene (650 ml) concentrated aq. HBr (48 %, 30 ml) was added. The mixture was heavily stirred and heated at reflux for 24 h whereupon additional HBr (48 %, 15 ml) was added and the mixture was refluxed another 24 h. The reaction mixture was allowed to cool to rt, and the phases were separated. The organic layer was washed with water (50 ml), saturated aq. NaHCO_3_ (50 ml), and brine (50 ml). Drying (MgSO_4_) and concentration gave a yellow oil of **5** (39.0 g) which was used in the next step without further purification.

2-((5-Bromopentyl)oxy-tetrahydro-2*H*-pyran (**6**) (Bongini et al. 1979)

The product from previous reaction was dissolved in dichloromethane (300 ml). A catalytic amount (ca. 500 mg) of Amberlyst^®^15 was added to the solution and followed by dropwise addition of 3,4-dihydro-2*H*-pyran (25.2 g, 300 mmol). The reaction mixture was stirred for 6 h at rt., the catalyst filtered off and solvent evaporated. The residue was purified by MPLC yielding **6** (52.8 g, 87.7 % after two steps).

^1^H NMR (400 MHz, CDCl_3_): *δ* (ppm): 4.58–4.50 (m, 1H, H-1′), 3.83–3.90 (m, 1H, H-5′), 3.80 (dt, *J* = 10.1, 6.3 Hz, 1H, H-1), 3.66 (t, *J* = 7.0, 2H, H-5), 3.46–3.53 (m, 1H, H-5′), 3.42 (dt, *J* = 9.7, 5.6 Hz, 1H, H-1), 2.17 (br.s., 1H, –OH), 1.45–1.87 (m, 12 H, H-2, H-3, H-4; H-2′, H-3′, H-4′).

### 2-((5-iodopentyl)oxy)tetrahydro-2*H*-pyran (**7**)

NaI (14.8 g, 98.6 mmol) was added portion wise to bromide **6** (16.5 g, 65.7 mmol) in dry acetone (200 ml) and the mixture was stirred at rt overnight. Precipitated NaBr was filtered off and the reaction mixture concentrated under vacuum. The resulting oil was purified by MPLC yielding **7** (18.4 g, 94.0 %). 1H NMR (400 MHz, CDCl_3_): *δ* (ppm): 4.59 - 4.54 (m, 1H, H-1′), 3.89–3.81 (m, 1H, H-5′), 3.74 (dt, *J* = 9.7, 6.6 Hz, 1H, H-1), 3.53–3.46 (m, 1H, H-5′), 3.38 (dt, *J* = 9.7, 6.4 Hz, 1H, H-1), 3.19 (t, *J* = 7.0 Hz, 2H, H-5), 1.44–1.90 (m, 12H, C2-H_2,_ C3-H_2_, C4-H_2_, C3′-H_2_, C4′-H_2_, C5′-H_2_).

### 2-((5-(3,4-dimethylfuran-2-yl)pentyl)oxy)tetrahydro-2*H*-pyran (**8**)

*n*-BuLi (21.2 mL 2.5 M solution in hexanes, 53 mmol) was added dropwise into the stirred solution of **4** (4.8 g, 50 mmol) in THF (150 mL) at −20 °C. Stirring was continued for 2 h at the same temperature before **7** (15.8 g, 53 mmol) in THF (20 mL) was added slowly. Stirring was continued for 2 h at rt. The reaction was quenched by adding H_2_O (150 mL). The aqueous phase was extracted with petroleum ether (3 × 100 mL), dried (MgSO_4_), and concentrated. Purification using MPLC yielded **8** (11.2 g, 84 %).

^1^H NMR (400 MHz, CDCl_3_): *δ* (ppm): 7.02 (d, *J* = 1.2 Hz, 1H, H-9), 4.56 (dd, *J* = 4.4, 2.9 Hz, 1H, H-1′), 3.90–3.81 (m, 2H, H-5′), 3.76–3.68 (m, 1H, H-1), 3.49 (dddd, *J* = 10.9, 5.3, 3.5, 1.2 Hz, 1H, H-5′), 3.42–3.34 (m, 1H, H-1′), 2.53 (t, *J* = 7.4 Hz, 2H, H-5), 1.91 (d, *J* = 1.2 Hz, 3H, H-8A), 1.85 (s, 3H, H-7A), 1.87–1.79 (m, 1H, H-3′), 1.75–1.68 (m, 1H, H-2′), 1.64–1.46 (m, 8H, H-2, H-4, H-2′, H-3′, H-4′), 1.41 - 1.34 (m, 2H, H-3).

^13^C NMR (101 MHz, CDCl_3_): δ (ppm): 151.2, 136.2, 120.9, 114.4, 98.8, 67.5, 62.3, 30.8, 29.5, 28.3, 26.2, 25.8, 25.5, 19.7, 8.4, 7.9.

### 3,4-dimethyl-5-(5-((tetrahydro-2*H*-pyran-2-yl)oxy)pentyl)furan-2-carbaldehyde (**9**)

*n*-BuLi (16.9 mL 2.5 M solution in hexanes, 42.1 mmol) was added dropwise to the stirred solution of **8** (10.2 g, 38.3 mmol) in THF (120 mL) at −5 °C. Stirring was continued for 2 h at rt, before DMF (4.5 g, 61.3 mmol) was added dropwise at 0 °C. Stirring was continued for 4 h at rt. The reaction was quenched by adding H_2_O (150 mL). The aqueous phase extracted with Et_2_0 (3 × 120 mL) was washed sequentially with 1 M HCl, saturated aq. NaHCO_3_ and brine. After being dried (MgSO_4_), concentrated and purified using MPLC, the yield of **9** was 7.0 g (62 %).

^1^H NMR (400 MHz, CDCl_3_): *δ* (ppm): 9.60 (s, 1H, aldeh.), 4.54 (dd, *J* = 4.6, 2.8 Hz, 1H, H-1′), 3.89–3.78 (m, 1H, H-5′), 3.71 (dt, *J* = 9.6, 6.7 Hz, 1H, H-1), 3.48 (dddd, *J* = 11.3, 5.6, 4.0, 1.3 Hz, 1H, H-5′), 3.36 (dt, *J* = 9.6, 6.5 Hz, 1H, H-1), 2.62 (t, *J* = 7.5 Hz, 2H, H-5), 2.25 (s, 3H, H-8A), 1.90 (s, 3H, H-7H), 1.79 (dddd, *J* = 8.1, 6.3, 4.5, 2.2 Hz, 1H, H-3′), 1.73–1.63 (m, 3H, H-4, H-2′), 1.63–1.44 (m, 6H, H-2, H-2′, H-3′, H-4′), 1.43–1.33 (m, 2H, H-3).

^13^C NMR (101 MHz, CDCl_3_): δ (ppm): 171.1, 158.7, 147.04, 147.02, 118.8, 98.7, 67.3, 62.3, 30.7, 29.4, 27.6, 26.5, 25.9, 25.5, 19.7, 8.8, 7.

### (*E*)-ethyl 3-(3,4-dimethyl-5-(5-((tetrahydro-2*H*-pyran-2-yl)oxy)pentyl)furan-2-yl)acrylate (**10**)

LiO*t*Bu (1 M solution in hexanes, 32 mmol) was added to a solution of triethyl 2-phosphonoacetate (9.74 g, 43.5 mmol) in dry hexane (30 mL) at 0 °C. The reaction mixture was stirred at rt for 1 h before **9** (8.63 g, 29.4 mmol) in Et_2_O (40 mL) was added dropwise. After being stirred overnight at rt, the reaction was quenched by adding H_2_O (100 mL). The aqueous phase was extracted with PE (3 × 60 mL). The combined organic phase was washed with 0.1 N NaOH (30 ml) and brine (30 mL), dried, and concentrated. Purification using MPLC yielded 12 (9.32 g, 88 %).

^1^H NMR (400 MHz, CDCl_3_): *δ* (ppm): 7.41 (d, *J* = 15.5 Hz, 1H, H-10), 6.13 (d, *J* = 15.5 Hz, 1H, H-11), 4.56 (dd, *J* = 4.5, 2.7 Hz, 1H, H-1′), 4.22 (q, *J* = 7.1 Hz, 2H, H-1″), 3.84 (ddd, *J* = 11.0, 7.5, 3.4 Hz, 1H, H-5′), 3.72 (dt, *J* = 9.6, 6.8 Hz, 1H, H-1), 3.52–3.45 (m, 1H, H-5′), 3.38 (dt, *J* = 9.6, 6.5 Hz, 1H, H-1), 2.56 (t, *J* = 7.4 Hz, 2H, H-5), 2.02 (s, 3H, H-8A), 1.85 (s, 3H, H-7A), 1.80 (td, *J* = 7.8, 6.5, 3.6 Hz, 1H, H-3′), 1.74–1.46 (m, 9H, H-4, H-2, H-2′, H4′, H3′), 1.44–1.35 (m, 2H, H-3), 1.30 (t, *J* = 7.1 Hz, 3H, H-2″).

^13^C NMR (101 MHz, CDCl_3_): δ (ppm): 167.9, 154.3, 144.8, 129.22, 127.6, 117.6, 111.6, 98.8, 67.4, 62.3, 60.0, 30.7, 29.4, 28.0, 26.3, 25.8, 25.5, 19.6, 14.4, 8.8, 8.0.

### Ethyl 3-{3,4-dimethyl-5-[5-(tetrahydrofuran-2-yloxy)pentyl]-2-furyl}propanoate (**11**)

Ester **10** (8.80 g, 24.2 mmol) was dissolved in hexane (170 mL) and this solution was purged with argon before the catalyst, 10 % of palladium on carbon (530 mg) was added, and hydrogen was bubbled into the reaction mixture. The mixture was stirred under the pressure of hydrogen. Starting material disappearance and the beginning of the formation of over-reduced compound (reduced double bonds of the tetrahydrofuran ring) were monitored by TLC. The reaction was stopped after 3 h by purging with argon. The catalyst was filtered through a pad of Celite. After solvent removal and purification by MPLC **11** (7.96 g, 90 %) was afforded.

^1^H NMR (400 MHz, CDCl_3_): *δ* (ppm): 4.57 (dd, *J* = 4.5, 2.7 Hz, 1H, H-1′), 4.13 (q, *J* = 7.1 Hz, 2H, H-1″), 3.86 (ddd, *J* = 11.0, 7.6, 3.4 Hz, 1H, H-5′), 3.72 (dt, *J* = 9.6, 6.9 Hz, 1H, H-1), 3.52–3.46 (m,1H, H-5′), 3.37 (dt, *J* = 9.6, 6.6 Hz, 1H, H-1), 2.83 (dd, *J* = 8.8, 6.7 Hz, 2H, H-10), 2.57 (dd, *J* = 8.8, 6.8 Hz, 2H, H-11), 2.48 (t, *J* = 7.4 Hz, 2H, H-5), 1.78-1,87 (m, 1H, H-3′), 1.84 (s, 3H, H-8A), 1.81 (s,3H, H-7A), 1.75–1.66 (m, 1H, H-2′), 1.65–1.47 (m, 8H), 1.41–1.33 (m, 2H, H-3, H-2, H-4, H-2′, H-3′, H4′), 1.24 (t, *J* = 7.1 Hz, 3H, H-2″).

^13^C NMR (101 MHz, CDCl_3_): *δ* (ppm): 172.9, 148.8, 145.9, 115.4, 114.7, 98.8, 67.5, 62.3, 60.3, 33.3, 30.8, 29.5, 28.5, 26.0, 25.8, 25.5, 21.7, 19.7, 14.2, 8.3, 8.2.

### Ethyl 3-(5-(5-hydroxypentyl)-3,4-dimethylfuran-2-yl)propanoate (**12**) (Coppola 1984)

To remove the protection from the hydroxyl group, the ester **11** (10.40 g, 28.4 mmol) in MeOH (50 mL) was stirred and heated at 55 °C in the presence of Amberlyst^®^15 as a catalyst for 5 h. The catalyst was filtered off, the solvent removed, and residue purified by MPLC to yield **12** (6.86 g, 86 %).

^1^H NMR (400 MHz, CDCl_3_): *δ* (ppm): 4.12 (q, *J* = 7.1 Hz, 2H, H-1″), 3.63 (t, *J* = 6.6 Hz, 2H, H-1), 2.83 (t, *J* = 6.7 Hz, 2H, H-10), 2.56 (t, *J* = 6.6 Hz, 2H, H-11), 2.49 (t, *J* = 7.3 Hz, 2H, H-5), 1.84 (s, 3H, H-8A), 1.81 (s, 3H, H-7A), 1.64–1.52 (m, 4H, H-2, H-4), 1.48 (s, 1H, -OH), 1.41–1.31 (m, 2H, H-3), 1.24 (t, *J* = 7.1 Hz, 3H, H-2″).

^13^C NMR (101 MHz, CDCl_3_): δ (ppm): 172.9, 148.7, 146.0, 115.4, 114.8, 62.9, 60.4, 33.3, 32.5, 28.4, 25.9, 25.3, 21.7, 14.2, 8.24, 8.21.

### 3-(5-(5-hydroxypentyl)-3,4-dimethylfuran-2-yl)propanoic acid (**13**)

Ester **12** (2.50 g, 8.87 mmol) was hydrolyzed by heating it at 45 °C with KOH (2.45 g) in 1:1 mixture of H_2_O and MeOH (80 mL) for 4 h. The solution was concentrated until half of the volume was left and H_2_O (40 mL) was added prior the extraction with PE (2 × 50 mL). The aqueous phase was acidified with 1 M HCl to pH 1 and free acid **13** extracted with Et_2_O (4 × 50 mL). The combined extracts were washed with brine (30 mL) and dried (MgSO_4_). Removing of the solvent continued until some Et_2_O was still present. The amount of **13** (2.20 g, 98 %) was estimated from NMR spectra.

^1^H NMR (400 MHz, CDCl_3_): *δ* (ppm): 3.65 (t, *J* = 6.3 Hz, 2H, H-1), 2.85 (t, *J* = 7.2 Hz, 2H, H-10), 2.60 (t, *J* = 7.2, Hz, 2H, H-11), 2.51 (t, *J* = 7.0 Hz, 2H, H-5), 1.84 (s, 3H, H-8A), 1.81 (s, 3H, H-7A), 1.63–1.50 (m, 4H, H-2, H-4), 1.40–1.30 (m, 2H, H-3).

^13^C NMR (101 MHz, CDCl_3_): *δ* (ppm): 177.0, 148.8, 145.7, 115.5, 115.0, 62.9, 32.9, 32.3, 28.3, 25.7, 25.3, 21.5, 8.24, 8.21.

### 12,13-dimethyl-5,14-dioxabicyclo[9.2.1]tetradeca-1(13),11-dien-4-one (**1**)

Diethyl azodicarboxylate (DEAD, 19.9 mL of 40 % solution in toluene, 45 mmol) was added to triphenylphosphine (11.35 g, 43.9 mmol) in toluene (120 ml) at rt and the mixture was stirred for 15 min. Hydroxy acid **13** (2.20 g, 8.66 mmol) in toluene (120 mL) was added over a period of 5 h. The reaction mixture was stirred for 3 h and stored overnight at −18 °C. After warming to the ambient temperature, the solvent was removed and PE (200 mL) was added to precipitate triphenylphosphine oxide. After being filtrated and purified two times by MPLC **1** was obtained (1.37 g, 67 %).

^1^H NMR (400 MHz, CDCl_3_): *δ* (ppm) (Fig. 7):

4.22–4.15 (m, 2H, H-1), 2.91–2.84 (m, 2H, H-10), 2.58 (t, *J* = 6.5 Hz, 2H, H-5), 2.43–2.37 (m, 2H, H-11), 1.84 (s, 3H, H-8A), 1.81 (s, 3H, H-7A), 1.70 (dq, *J* = 10.9, 5.8 Hz, 2H), 1.60 (p, *J* = 6.6 Hz, 2H, H-4), 1.24–1.14 (m, 2H, H-3).

^13^C NMR (101 MHz, CDCl_3_) ?

173.7, 148.3, 146.2, 115.2, 115.1, 63.0, 34.9, 25.3, 25.0, 23.3, 22.8, 22.2, 8.2, 8.0.
